# Mental and Substance Use Disorders Prevalence Study: Background and Methods

**DOI:** 10.1002/mpr.2000

**Published:** 2024-01-22

**Authors:** Heidi Guyer, Heather Ringeisen, Jill Dever, Dan Liao, Andy Peytchev, Christine Carr, Paul Geiger, Leyla Stambaugh, Tim Smith, Lisa Dixon, Mark Olfson, Michael First, Scott Stroup, Lydia Chwastiak, Maria Monroe‐Devita, Jeff Swanson, Marvin Swartz, Ronald C. Kessler, Robert Gibbons, Natalie Bareis, Elizabeth Sinclair Hancq, Thomas Clarke, Mark Edlund, the MDPS Consortium, Thomas Smith, Mackenzie Tennison, Katherine Winans, Scott Graupensperger, Allison Robertson

**Affiliations:** ^1^ RTI International Research Triangle Park North Carolina USA; ^2^ Columbia University/New York State Psychiatric Institute New York New York USA; ^3^ University of Washington Seattle Washington USA; ^4^ Duke Health Durham North Carolina USA; ^5^ Harvard Medical School Cambridge Massachusetts USA; ^6^ University of Chicago Chicago Illinois USA; ^7^ Treatment Advocacy Center Arlington Virginia USA; ^8^ Substance Abuse and Mental Health Services Administration (SAMHSA) Rockville Maryland USA

**Keywords:** clinical interview, epidemiologic research design, prevalence, psychiatric epidemiology, SCID‐5

## Abstract

**Objective:**

The Mental and Substance Use Disorders Prevalence Study (MDPS) builds upon previous epidemiologic studies to provide estimates of prevalence and treatment rates of mental and substance use disorders among adults aged 18–65 in the U.S. The study background and methods are described.

**Method:**

The MDPS employed novel techniques such as the inclusion of household, prison, homeless and state psychiatric hospital populations, a semi‐structured clinical interview administered by trained clinical interviewers to assess disorders, the assessment of both past year and lifetime schizophrenia spectrum disorder (SSD) using full Diagnostic and Statistical Manual 5 criteria, as well as other mental and substance use disorders, and video‐based interviewing. Population specific and combined sample weights were developed to estimate nationally representative prevalence and treatment rates.

**Results:**

Data collection was conducted between October 2020 and October 2022 resulting in 5679 clinical interviews. The statistical weighting and analytic plan are described. Weighted response rates and reasons for non‐response are provided for each study population.

**Conclusions:**

The MDPS successfully developed and employed novel techniques to estimate the prevalence and treatment rates of mental and substance use disorders in both household and non‐household populations, including some of the most impairing disorders such as SSD.

## 1 INTRODUCTION: BACKGROUND AND AIMS

This paper describes the sampling, data collection, instruments, statistical weighting, and analytical plan for the Mental and Substance Use Disorders Prevalence Study (MDPS). The MDPS provides the most up‐to‐date prevalence estimates of specific mental health disorders in the non‐elderly U.S. adult population. The three key aims of the MDPS are to (1) provide national prevalence estimates of mental health and substance use disorders (SUDs) among U.S. adults ages 18–65, (2) determine what proportion of individuals with mental and SUDs received any treatment in the past 12 months, and (3) investigate research methods for improving future studies like the MDPS. The study provides lifetime and past‐year prevalence rates of schizophrenia spectrum disorders (SSDs, defined as including schizophrenia, schizoaffective disorder, and schizophreniform disorder) and past‐year prevalence rates for bipolar I disorder, major depressive disorder, generalized anxiety disorder (GAD), posttraumatic stress disorder (PTSD), obsessive‐compulsive disorder, anorexia nervosa, and past‐year alcohol, opioid, cannabis, stimulant, and sedative/hypnotic/anxiolytic use disorders (Ringeisen et al., 2023).

The MDPS builds on the history and contributions of prior U.S. and international psychiatric epidemiology studies. The Diagnostic and Statistical Manual of Mental Disorders, Third Edition (American Psychiatric Association, 1980) was the first DSM edition to provide specific criteria for diagnoses. This made possible large‐scale psychiatric epidemiological studies in the United States. DSM‐III diagnostic criteria could be operationalized into fully structured interviews and delivered by lay interviewers (i.e., not mental health clinicians) to assess disorder prevalence rates. The first fully structured interview using the DSM‐III was the Diagnostic Interview Schedule (DIS) developed by Lee Robins and colleagues (Helzer & Robins, 1988; Robins et al., 1981; Singerman et al., 1981). Coinciding with the development of the DSM‐III, increasingly sophisticated methods were being developed for community surveys. In the early 1980s investigators brought together these two methodologies—fully structured interviews conducted by lay interviewers and community surveys—in the Epidemiological Catchment Area (ECA) study (Narrow et al., 1993; Regier et al., 1993; Robins & Regier, 1990). In the ECA, over 20,000 individuals living in five cities (Baltimore, MD; Durham, NC; Los Angeles, CA; New Haven, CT; and St. Louis, MO) were interviewed twice, 1 year apart, using the DIS. These interviews were conducted face to face in households, which would have been prohibitively expensive and infeasible with mental health clinicians, and with respondents in prisons and hospitals. Future psychiatric epidemiological studies built on the ECA including the National Comorbidity Study (Kessler et al., 1994), the National Comorbidity Survey Replication (NCS‐R; Kessler et al., 2004; Kessler, Berglund, et al., 2005; Kessler, Birnbaum, et al., 2005) and World Health Organization (WHO) World Mental Health Studies (Kessler & Ustün, 2004, 2008), the three waves of the National Epidemiological Survey of Alcohol Related Conditions (NESARC; Hasin & Grant, 2015; Hasin et al., 2018), and the National Survey on Drug Use and Health (NSDUH; Substance Abuse and Mental Health Services Administration, 2020). Of these, only the ECA provided catchment area estimates of SSDs and included both household and nonhousehold (prison, hospital) participants (Hasin & Grant, 2015).

These studies established the descriptive epidemiology of mental health and SUDs, and important patterns have emerged. Mental and SUDs occur commonly and are often comorbid (e.g., Hasin & Grant, 2015; Kessler et al., 1996, Kessler, Birnbaum, et al., 2005). Onset typically occurs in late adolescence or early adulthood, and earlier age of onset is often correlated with greater disorder severity and disability (e.g., Kessler, Chiu, et al., 2005). The prevalence of depression and anxiety disorders is higher among females than males (e.g., Hasin & Grant, 2015; Kessler, Birnbaum, et al., 2005), while the prevalence of SUDs is more common among males than females (e.g., Hasin & Grant, 2015; Substance Abuse and Mental Health Services Administration, 2020). There is high unmet need for treatment, with approximately 50% of receiving no treatment at all (e.g., Olfson et al., 2019). Further, among individuals who receive any treatment, many do not receive treatment consistent with recommended best practices for their disorder (e.g., Grant et al., 2005; Kessler, Birnbaum, et al., 2005; Young et al., 2001).

### 1.1 Unique features of the MDPS design

Compared to prior U.S. national psychiatric epidemiological studies, the MDPS has four unique features: (1) the measurement of lifetime and past‐year SSDs, (2) the administration of semi‐structured clinical interviews conducted by mental health clinicians, (3) the use of a multistage household survey design (roster, screening, clinical interview), and (4) the inclusion of household and non‐household sample populations. As explained below, the motivation for the last three design choices stemmed from the desire to optimize measurement of SSDs.

First, the MDPS assesses SSDs using full DSM criteria, which prior studies have not done. SSDs are highly impairing. In the WHO Global Burden of Disease study, acute schizophrenia had a higher disability weight (0.788) than severe multiple sclerosis (0.732) or untreated spinal cord lesion in the neck (0.719; Salomon et al., 2015; in this study, 0 represented no loss of health, and 1 was loss equivalent to death). Further, schizophrenia is associated with a decrease in life expectancy of 10–20 years (e.g., Hjorthoj et al., 2017; Kyu et al., 2018).

The assessment of SSDs requires use of a semi‐structured clinical interview rather than a fully structured interview conducted by lay interviewers. DSM‐5 SSD diagnostic criteria are based on both patient report (delusions, hallucinations) and clinical observations (negative symptoms, grossly disorganized or catatonic behavior, and disorganized speech). In contrast, the diagnostic criteria of almost all other DSM disorders are based solely on patient self‐report. Fully structured interviews conducted by lay interviewers, commonly used in prior epidemiological studies, cannot assess those diagnostic criteria which require clinical observation. For example, lay interviewers on the ECA used the highly structured DIS and were asked to record their observations on the respondents reliability but not make inferences about other symptoms or disorders, such as psychotic disorders. Therefore, the MDPS utilized the Structured Clinical Interview for DSM‐5 (SCID‐5®; First et al., 2015) delivered by video teleconference to facilitate observations. The SCID‐5 is conducted by trained mental health clinicians and is the gold standard for mental and SUD diagnostic assessment. In contrast past studies have utilized a fully structured interview, conducted by lay interviewers for the diagnostic assessment, and used the SCID in validation studies of the diagnostic assessment.

The MDPS included a multistage household design to identify persons likely to have an SSD or other disorder for enhanced data collection via a clinical interview. This design featured a household roster to identify age‐eligible adult residents (from which at most two adults were randomly selected for the MDPS), a screening interview to assess the respondent's level of risk for mental disorders, and a clinical interview to measure specific mental and SUDs. Individuals identified at screening as having an elevated risk for SSDs were selected at 100% for the clinical interview; individuals identified at screening as having low risk for mental disorders were selected at a much lower rate. This multistage design resulted in an enriched MDPS household clinical interview sample comprising more cases meeting criteria for mental disorders, particularly SSDs, than would have been achieved without screening. The enriched clinical interview sample also helped increase analytic power to meet the study's second objective—to determine what proportion of individuals with mental and SUDs received any treatment in the past 12 months.

The fourth unique feature of the MDPS was the inclusion of household, prison, homeless, and state psychiatric hospital populations. We term the last three populations collectively as “non‐household populations.” One MDPS study objective was to investigate research methods for use in future studies. Key to this objective was understanding how to successfully implement high‐quality MDPS‐like data collection methods in non‐household settings. Prior psychiatric epidemiological studies have often excluded institutionalized populations such as people who are incarcerated, the homeless, and patients in state psychiatric hospitals. These individuals are at high risk for mental health and SUDs (e.g., Ayano et al., 2019), and their exclusion could systematically bias prevalence estimates downward. To achieve better national representation, and thus generalizability, the MDPS design included these populations in its sample. The MDPS study included more non‐household cases than would be necessary for national population representation to adequately support the study's third methodological objective. We note that the ECA included prison and nursing home populations, and the NSDUH includes the homeless.

### 1.2 Study overview

The MDPS is a 4‐year cooperative agreement between the Substance Abuse and Mental Health Services Administration (SAMHSA) and RTI International (Federal Award H79FG000030). RTI leads this study in partnership with Columbia University and the New York State Psychiatric Institute, University of Washington, Duke University School of Medicine, Harvard University, University of Chicago, Treatment Advocacy Center, TeleSage, and Adaptive Testing Technologies.

The project began in September 2019. Data collection was conducted between October 2020 and October 2022, during the coronavirus disease 2019 (COVID‐19) pandemic. The initial design and study protocol were developed prior to the abrupt COVID‐19 onset, necessitating significant changes and adaptations to the original protocol. These modifications are described throughout this paper. Perhaps most importantly, to reduce the impact of the COVID‐19 pandemic on data collection, MDPS data collection also occurred by web, mail and phone for household enumeration and mental health screening, and by video and phone for the clinical interviews, rather than relying solely on in‐person data collection. For example, 66.7% of the household interviews were conducted by video (Zoom), and the other 33.3% were conducted by phone (Table 1).

**TABLE 1:** Mode of data collection by instrument in the household population.

	Roster		Screener		Clinical interview	
	Count	Percent	Count	Percent	Count	Percent
Web	16,622	64.5	20,753	71.4	*na*	
Paper	2146	8.3	111	0.4	*na*	
Telephone	735	2.9	2363	8.1	1586	33.3
In‐person	6249	24.3	5857	20.1		
Virtual	*na*		*na*		3178	66.7

Abbreviation: *na*, data collection mode is not applicable for the instrument.

MDPS study protocols, instruments, and consent forms were reviewed and approved by the Advarra Institutional Review Board. RTI and all partner sites entered into reliance agreements with Advarra.

This paper describes the MDPS sample design and data collection for household and non‐household populations, the instruments, clinical interviewer (CI) training and quality assurance, and the study's responsive design. The paper also summarizes the statistical methods used to generate survey analysis weights, the nonresponse bias analyses, and the statistical methods to generate national prevalence and treatment estimates using the combined population‐specific information.

## 2 STUDY METHODS

### 2.1 Sampling

The core of the MDPS was a large national probability‐based household sample, supplemented with smaller samples from three non‐household populations: state/federal prisons, state psychiatric hospitals, and homeless shelters. Including these non‐household populations provides more complete coverage of the MDPS target population—U.S. adults aged 18–65. The upper age bound of 65 years was because of the emphasis on accurately assessing rates of SSDs, that is, primary psychoses. Individuals over 65 are at a significantly higher of risk of dementia and delirium, which is sometimes associated with psychosis. In a cross‐sectional interview with no potentially relevant past medical history available to interviewers, it could be difficult to differentiate these psychoses from SSDs in older adults.

### 2.2 Household sample

Household data collection was conducted between October 2020 and July 2022. The initiation of data collection was not postponed because of COVID‐19. Originally, data collection was to have taken 1 year but the end date was extended because of recruitment difficulties stemming from the pandemic. The household sample was drawn via a stratified, multistage sampling scheme. The first stage of sample selection included 100 primary sampling units (PSUs) defined as counties or groups of counties. Originally we planned to use 50 PSUs, but with the COVID‐19 pandemic it became clear that household roster data collection would be mainly, if not entirely, virtual, as opposed to in person as specified in the original study protocols. Because of the decreased need for in‐person field work, we increased the number of PSUs to 100 to decrease the variance of estimates. Then in the second stage, 16 secondary sampling units (SSUs), defined by census block groups, were selected within each PSU. In the third and final stage, households were selected within each SSU via address‐based sampling (The American Association of Public Opinion Research, 2016). The household sample was released in sets of sample replicates (groups) to capture efficiencies in real time for subsequent efforts. A small pilot was conducted between October and December 2020 with the first replicate. The second larger replicate of household addresses was released in January 2021. Over the course of data collection, household sample replicates were released nine times, and each replicate included between 16,000 and 43,735 eligible addresses each time. In total, 234,270 household addresses were released. We had originally planned to release a total of 37,037 household addresses, but the inability to conduct in‐person household rostering lowered our response rate (RR), requiring us to increase the sample released to 234,270 to achieve our final target goal of completed interviews in the household sample.

**FIGURE 1:**
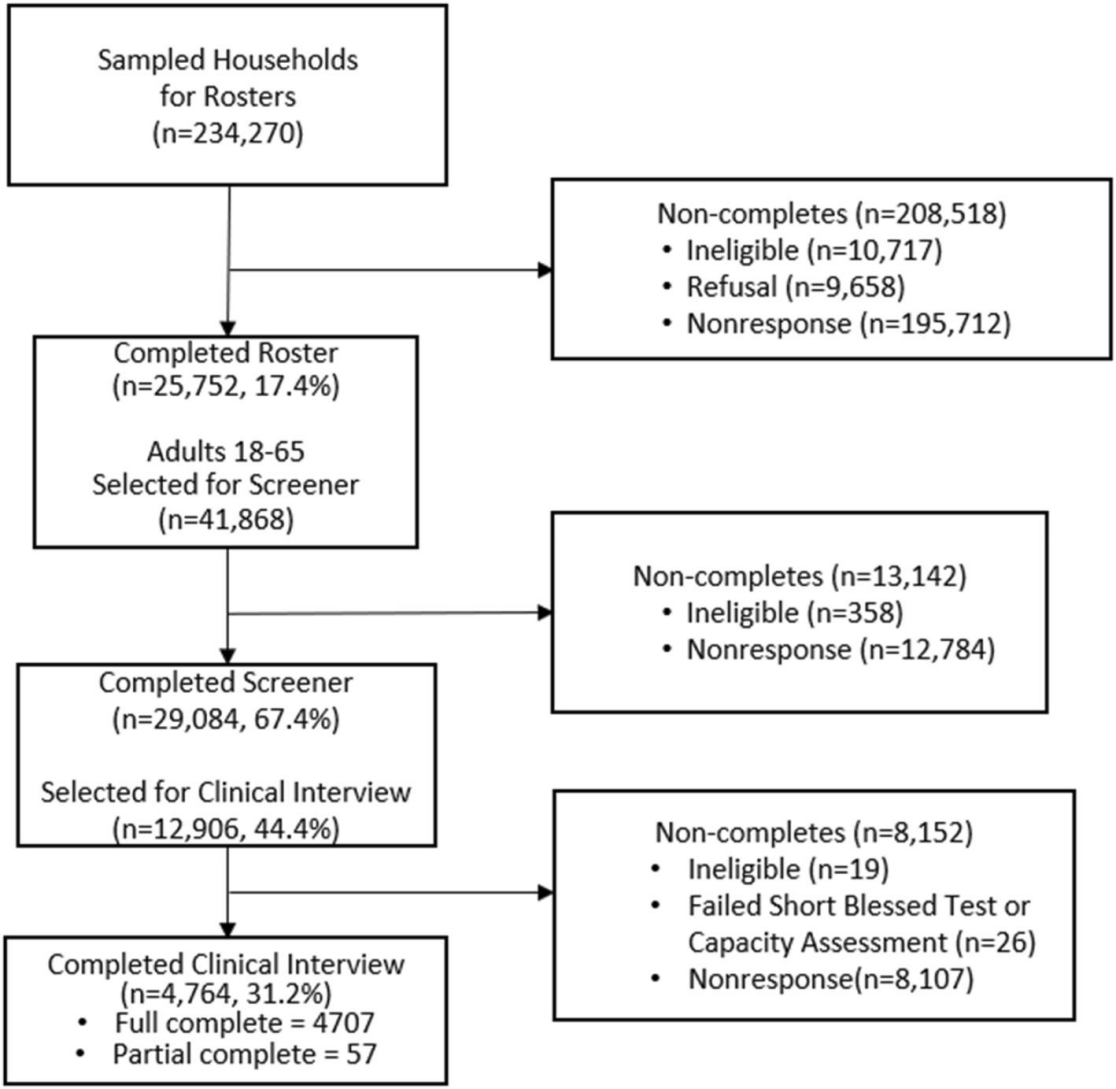
Household sample diagram and weighted response rates.

An overview of the steps in household data collection is shown in Figure 1. Each sampled household was sent a letter explaining the study and offering the option to complete the roster via web, telephone, or mail. A household member at the sampled address completed a roster to determine study eligibility. The roster requested a listing of all adult household members, their age (which determined eligibility), contact information, and whether any household member had one or more chronic health conditions (e.g., heart problems, schizophrenia). To improve RRs, some nonresponding households in earlier sample release replicates were selected and followed up via in‐person interviewing to complete the roster beginning in May 2021. Approximately 37% of the household sample was released to trained field interviewers for in‐person rostering. This resulted in approximately one‐quarter of all rosters being completed in person with the remaining 75% completed by web, mail, or phone.

If one or two age‐eligible individuals lived in the rostered household, each adult was invited to participate in a subsequent screening interview. If three or more age‐eligible individuals lived in the household, two were randomly selected to participate in the screening interview. This interview screened for individuals at higher risk of SSDs and other mental or SUDs. Individuals who completed the screening interviews were randomly selected for the clinical interview via stratified sampling based on their screening responses so that those endorsing psychotic symptoms or other mental disorders had a higher selection probability. Trained clinicians (e.g., social workers and psychologists) conducted semi‐structured interviews using the SCID‐5 to assess mental and SUDs.

Completed screening interviews were used to classify the respondents into one of three hierarchical mental health risk strata: Stratum 1—those who reported experiencing psychotic symptoms or receiving disability payments because of schizophrenia, Stratum 2—those who reported experiencing symptoms of other MDPS disorders (e.g., major depressive disorder, GAD, alcohol use disorder), and Stratum 3—those who reported no symptoms associated with these mental or SUDs. One hundred percent of respondents from Stratum 1 were selected (i.e., all invited to complete a clinical interview). The selection rates for the other two strata were adjusted during the study to meet clinical interview study objectives, including the analytic goal of obtaining a target number of participants. Specifically, over the course of study data collection the sampling rate for Stratum 2 was increased from 20% to 80% and the sampling rate for Stratum 3 was increased from 8% to 20%. This sampling strategy helped increase the number of adults completing clinical interviews who might meet criteria for a mental or SUD, especially SSDs, allowing us to reach our interview targets. This procedure also provided an enriched MDPS sample designed to reduce the variance and increase the precision of prevalence rate estimates calculated with the clinical interview data.

### 2.3 Non‐household sample

**FIGURE 2:**
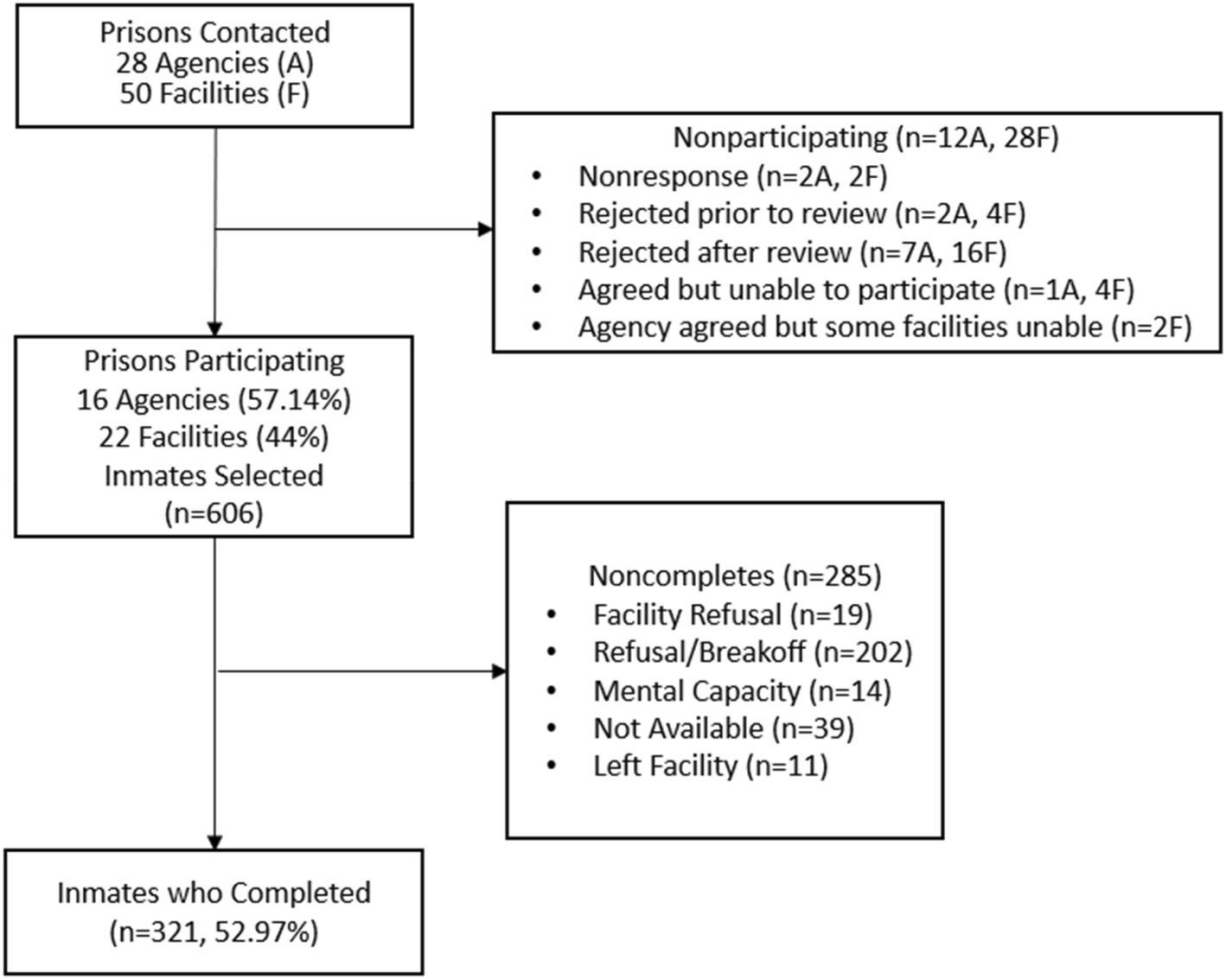
Prison flowchart.

**FIGURE 3:**
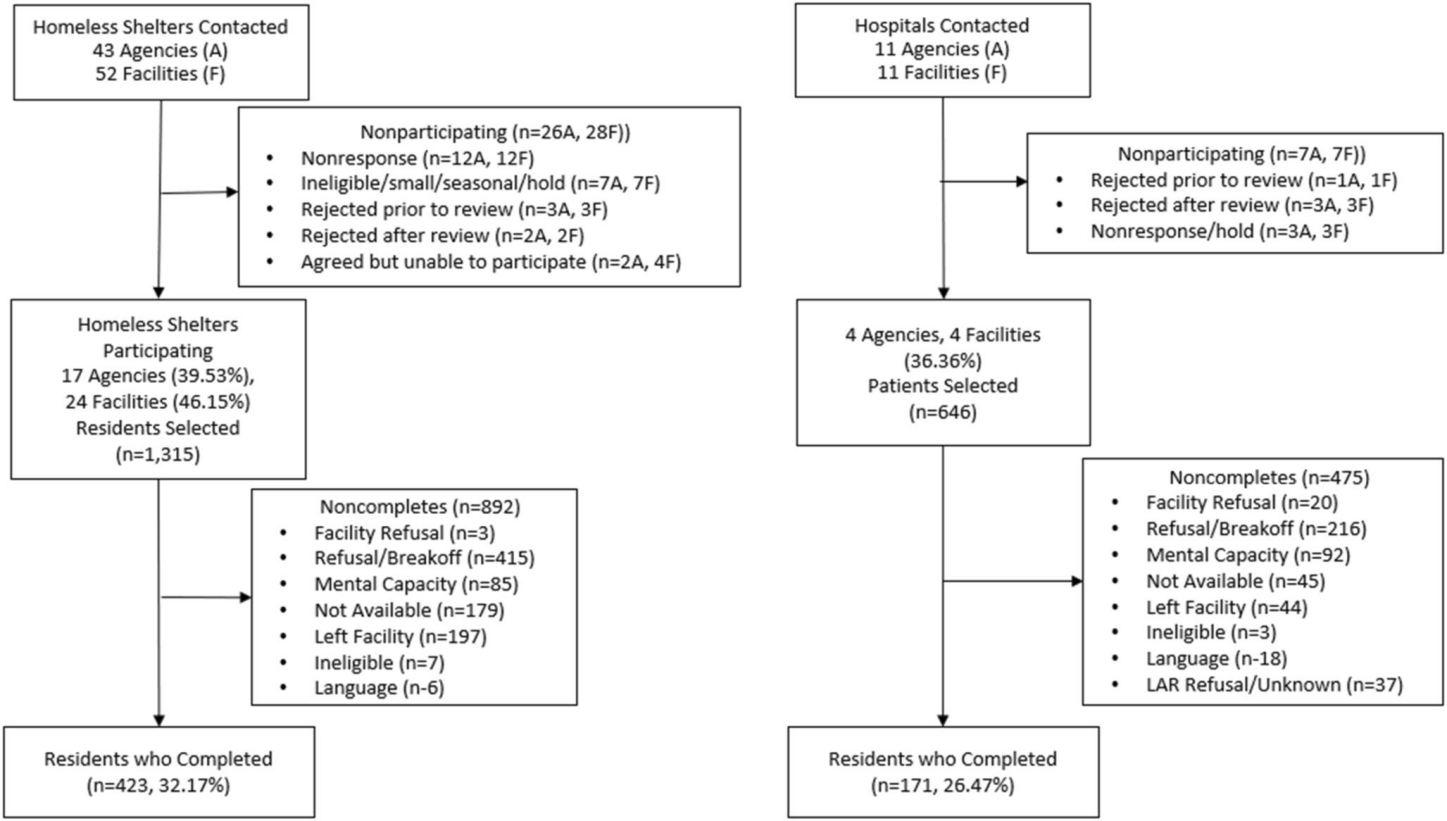
Hospital and homeless shelter flowcharts.

The MDPS non‐household sample included individuals residing in prisons, homeless shelters, and state psychiatric hospitals (Figures 2 and 3). Among the non‐household samples, 50 prisons were selected via a probability sampling scheme from a national list of prisons provided by the Bureau of Justice Statistics (BJS). Twenty‐two prisons consented to participate in the study. Prisons from the states with the largest prison populations declined participation, largely because of the COVID‐19 pandemic. Residents from four state psychiatric hospitals (in four states) and 24 homeless shelters (in five states) were recruited as convenience samples. The shelters were intentionally chosen from approximately 200 candidate shelters to encompass a diverse range of factors including geographical location, gender (for female‐only, male‐only, or family‐only), number of beds, and public versus private ownership. The household roster and screening interview were not used in the non‐household samples. A screener was not felt to be necessary because of the expected high rates of SSDs and other disorders in the non‐household samples relative to the household population and because it would add logistical complexity for the participating facilities.

Participating prison and hospital facilities submitted a roster of current individuals meeting the study eligibility criteria (e.g., aged 18–65). The roster was sorted by key characteristics of the individuals, such as age and time since admission. A sample was then selected from the sorted roster via a systematic sampling scheme. This implicitly stratified sampling technique was used to ensure that each selected non‐household sample was balanced by the characteristics used in this sorting (see, e.g., Valliant et al., 2018). Participating shelters could provide either a roster of their residents or the number of beds in lieu of a roster. For study recruitment, field staff selected all the residents in small shelters and selected a systematic sample of residents or beds in large shelters. Overall, non‐household populations were oversampled compared to the household population, meaning individuals in these non‐household populations had a higher chance to be selected in MDPS in comparison to individuals in the household population. One objective of the MDPS study was to investigate methods for use in future studies, including testing whether MDPS data collection methods could be implemented with high quality in these facility settings. As a result, more cases were selected from non‐household settings than would be necessary to represent the U.S. adult population. The disproportionate rate of non‐household case selection was subsequently addressed by statistical weighting when producing national estimates (see below).

Non‐household recruitment activities began in July 2020 and continued through September 2022. This included emails, phone calls, and hardcopy mailings to facility administrators and research coordinators; submission of study protocols for review and approval by the governing bodies of the facilities; and ongoing community engagement including study webinars and quarterly newsletters. Interviewing of facility residents began in March 2021 and continued through October 2022.

### 2.4 Modes of data collection

The MDPS was originally planned as a multimode design at each stage of data collection, including self‐ and in‐person administration. However, the onset of the COVID‐19 pandemic and the resulting stoppage of in‐person data collection on many national surveys required the addition of more data collection modes, enhancing non–in‐person contacting protocols, and increasing incentives. The study maximized the percentage of rostered households and screenings completed online and by phone, by starting with mailed invitations and sequentially adding options to complete the household rostering and screening on paper and, for early sample release replicates, in person. To aid participation rates, incentives were used at each stage: a $2 prepaid and $10 promised household roster incentive, a $20 promised household screening incentive, and a $30 clinical interview incentive. Clinical interviews were conducted either by video or phone with the household sample and by video, phone, or in person in the facility settings. CIs used a laptop and tablet computers to contact respondents and conduct the video‐based computer‐assisted personal interview. The count and percentage who completed the roster, screener, and clinical interview via each mode are shown in Table 1 (household) and Table 2 (non‐household).

**TABLE 2:** Mode of data collection for non‐household facilities.

Study population	Number of facilities	Phone interviews	Video interviews	In‐person interviews	Total interviews
State psychiatric hospital	4	2	50	119	171
Homeless shelter	24	13	107	303	423
Prison	22	16	123	182	321
Total	50	31	280	604	915

## 3 INSTRUMENTS

The MDPS utilized three instruments: (1) the household roster, (2) the household screening interview (which used one of two screening protocols), and (3) the semi‐structured clinical interview. All instruments were available in both English and Spanish.

### 3.1 Household roster

The household roster identified eligible individuals residing in sampled households. The roster collected only information needed to identify eligible members of the household between the ages of 18 and 65. The household roster administration time was 7.7 min on average (median = 6.4).

### 3.2 Household screening interview

The screening interview helped the research team oversample individuals at increased likelihood of having mental and SUDs. Large population surveys have not screened for specific mental disorders. Therefore, the MDPS compared two screening interviews—one adaptive and one non‐adaptive. Individuals were randomly selected to receive one of these two screening interviews for online administration; the non‐adaptive instrument was solely used for paper administration.

The first screening interview used items from the CIDI^®^ (Kessler & Ustün, 2004), developed by the WHO (Direk et al., 2010) and used in the NCS‐R study (Kessler et al., 2004). It included items assessing depression, GAD, mania, PTSD, psychosis, and SUDs. These screening items were not adaptive; the same items were administered to every respondent regardless of mode. The average (online) CIDI screening interview time was 15.1 min (median = 12.6).

The second screening interview, the Computerized Adaptive Test—Mental Health (CAT‐MH^®^), is a brief adaptive test of mental disorders (Gibbons et al., 2016). The CAT‐MH modules used in the MDPS are quantitative measures of the severity of depression (Gibbons et al., 2012), anxiety (Gibbons et al., 2014), mania/hypomania (Achtyes et al., 2015), PTSD (Brenner et al., 2021), psychosis (Guinart et al., 2020), and SUDs (Gibbons et al., 2020). These quantitative severity measures are predictive of underlying disorders such as major depressive disorder, GAD, bipolar I disorder, PTSD, schizophrenia, and SUDs, and have all been validated. The adaptive approach of the CAT‐MH uses the participant's answers to initial items to determine which items are subsequently administered. Maximally informative follow‐up items are selected from a large (1461 symptom‐items) “bank” of items to efficiently complete the assessment with as little response burden as possible. The average CAT‐MH screening interview time was 18.3 min (median = 16.3).

The disorders assessed were generally specified in the funding opportunity announcement (FOA) from SAMHSA (FOA FG‐19‐003), although we did add GAD to the SAMHSA list. Other disorders, while clinically important, such as panic disorder, were not added, due to concerns regarding respondent burden. Each of the screening interviews also included items, in addition to the CIDI and CAT‐MH, such as those to assess demographic characteristics, exposure to COVID‐19, and veteran status. Screening interview times account for administration of both CIDI/CAT‐MH and these supplemental items. Informed consent was obtained at the start of the screening interview.

### 3.3 Semi‐structured clinical interview

Previous studies use fully structured interviews delivered by lay interviewers to assess symptoms of mental and SUDs. In contrast, the MDPS used the SCID‐5 (First et al., 2015), a semi‐structured clinical interview for psychiatric diagnosis designed to be delivered by trained clinicians (e.g., social workers, psychologists, and psychiatrists) who are experienced with diagnostic interviewing and the DSM‐5. The SCID‐5 and its previous versions have very good to excellent reliability and validity (Gerdner et al., 2015; Lobbestael et al., 2011; Osório et al., 2019; Shankman et al., 2018; Zanarini et al., 2000; Zanarini & Frankenburg, 2001). The MDPS used a computerized version of the SCID‐5, the NetSCID (Brodey et al., 2016). In a validation study, researchers found that the NetSCID reduced both data entry and branching errors when compared to the paper version of the SCID‐5 (Brodey et al., 2016; First et al., 2015).

We worked with the authors of the SCID‐5 to tailor a population‐based version specifically for the MDPS to simplify the instrument and shorten its administration time. This version is termed the SCID‐5‐NSMH (National Study of Mental Health). The SCID‐5‐NSMH assessed the past‐year prevalence of disorders in Table 3. Lifetime prevalence was also assessed for SSDs. The MDPS study focus was on SSDs, irrespective of accompanying mood disturbances. So, the SCID‐5‐NSMH did not differentiate schizoaffective disorder from schizophrenia or from schizophreniform disorder. To do so would have required assessing lifetime major depressive episodes (MDEs), rather than past‐year MDEs. This change significantly decreased the clinical interview administration time. Because of this, the SCID‐5‐NSMH assessed the past‐year and lifetime prevalence rates of SSDs with symptoms' duration of 6 months or greater (i.e., schizophrenia and schizoaffective disorder) and SSDs with symptoms' duration of less than 6 months (i.e., schizophreniform disorder). Mood disorders (i.e., depression, bipolar) were differentiated from schizoaffective disorder.

**TABLE 3:** Mental and substance use disorders measured by the SCID‐5‐NSMH.^a^

Schizophrenia spectrum disorders (SSD)	Alcohol use disorder
Schizophrenia spectrum disorders (SSD)–Lifetime	Opioid use disorder
Major depressive disorder (MDD)	Stimulant use disorder
Generalized anxiety disorder (GAD)	Sedative/hypnotic/anxiolytic use disorder
Bipolar I disorder	Cannabis use disorder
Posttraumatic stress disorder (PTSD)	
Obsessive compulsive disorder (OCD)	
Anorexia nervosa	

^a^ Past year unless specified otherwise.

The clinical interview also included items assessing sociodemographic characteristics, cigarette and e‐cigarette use, suicidal ideation and behavior, housing stability and supports, treatment (inpatient, outpatient, and use of medications) for mental and SUDs, and disability status (e.g., receipt of Supplemental Security Income/Social Security Disability Insurance). The study included items to assess impacts of COVID‐19 on access to mental and SUDs treatment and medical care.

The prison version of the SCID‐5‐NSMH omitted the module on substance use. This avoided the possibility of a participant disclosing information that could lead to being charged with an infraction of prison rules. Other modifications were made to the clinical interview administered to the prison sample to increase the applicability of certain items for this population. For example, mania module items were changed to include examples feasible within that context (i.e., replaced “…did you do reckless things, like drive dangerously, or drink or use drugs without caring about the consequences” with “…did you do reckless things, like pick fights or ignore prison rules without caring about the consequences?”).

Interviewers were trained to focus the interview and to control digressions, with a special focus on the non‐household populations, given the space and time constraints in these settings. The average interview length varied by study population. The interview length within the prison sample was capped at 60 min because of logistics within the facilities, resulting in a lower average clinical interview length than the other study population samples. Average administration times for the full interview, including the SCID‐5‐NSMH and other clinical interview content, for each of the four study populations are shown in Table 4.

**TABLE 4:** Mean and median clinical interview duration (minutes) for the full interview and the SCID content by study population.

	Full clinical interview^a^		SCID‐5‐NSMH portion	
	Mean	Median	Mean	Median
Household	75.9	69.1	57.8	51.3
Prison	59.2	57.7	40.6	37.3
Homeless shelter	72.4	66.4	53.7	48.9
State psychiatric hospital	70.9	66.7	50.9	46.7

Abbreviation: NSMH, National Study of Mental Health.

^a^ The full clinical interview included the SCID‐5‐NSMH and modules to collect sociodemographic and other relevant characteristics.

### 3.4 Informed consent and ability to provide consent

Informed consent was obtained before each phase of interviewing. A knowledge check was administered prior to conducting the clinical interview to ensure that participants were informed and understood the purpose of the study, could refuse to answer any question, and had the opportunity to ask questions. The knowledge check included questions like “*True or False: You can refuse to answer any questions.*” If the participant was unable to correctly answer the knowledge check questions after several attempts, it was determined that the participant lacked the ability to consent, and the interview ended. Additionally, the Short Blessed Test was administered by the CI if it appeared that the participant did not understand the questions, provided conflicting information, or was unable to complete the interview on their own (Davis et al., 1990; Katzman et al., 1983). If the participant did not pass the Short Blessed Test the interview ended. In total, 181 (26 household, 155 non‐household) such interviews were terminated, and results from these interviews were not used for analyses.

## 4 DATA COLLECTION MANAGEMENT AND FIELDWORK ORGANIZATION

### 4.1 Field interviewers

The household roster and screening interviews were originally planned for in‐person administration by trained field interviewers visiting the selected household addresses. However, the COVID‐19 pandemic led to travel restrictions, which impacted in‐person data collection. Early in the field period (March 2021), a team of eight telephone interviewers were hired and trained to complete roster and screener interviews by phone. Beginning in May 2021, the study launched an in‐person household rostering effort for the early release samples only; this effort was significantly smaller than originally planned. Monthly training sessions were held with field interviewers as locations began opening up for in‐person field work. Safety protocols were implemented for field staff to ensure social distancing, adherence to masking and vaccine requirements, and other measures to minimize the likelihood of COVID‐19 exposures. Between May and August 2021, a total of 158 field interviewers were trained to conduct in‐person household rosters and screeners using a computerized instrument on a tablet computer. A final training was held in January 2022 to account for attrition and unstaffed areas. Field interviewers completed household rosters in English and Spanish. These interviewers also directed respondents to the MDPS website to complete roster and screening instruments online if preferred. Given time and resource constraints, only a subset of the household sample was released for in‐person work. Approximately 70,000 addresses from sample releases 1 and 2 were released for in‐person follow‐up, but field interviewers were only able to attempt contact for a fraction of these. Over 6000 households completed an in‐person roster with an MDPS field interviewer between May 2021 and June 2022. Interviewer‐assisted household screeners were completed during this time as well. All eligible adults selected for a clinical interview were then called by a CI to schedule the MDPS clinical interview. Table 1 shows the mode of completion for each household instrument.

### 4.2 MDPS clinical interviewers and clinical supervisors

MDPS required a team of CIs with clinical assessment experience to conduct clinical interviews. Clinical supervisors (CSs) provided supervision to five to eight CIs to ensure interview quality.

#### 4.2.1 Recruitment and hiring

CI and CS recruitment began in the summer of 2020. Minimum qualifications included a degree and training in a relevant field (e.g., psychology, psychiatry, social work) and clinical experience administering the SCID‐5, with priority given to applicants with prior SCID‐5 experience. Generally, sending job ads to directors of American Psychological Association (APA)‐accredited clinical and counseling doctoral programs yielded higher quality candidates than employment websites (e.g., Indeed, Monster Jobs, LinkedIn). Hiring in region‐specific areas was not prioritized because of the emphasis on virtual video‐based interviewing and the need for teams of interviewers to travel to facilities for non‐household interview sessions. A formal interview process was developed that included confirmation that applicants met basic education and experience requirements; telephone screening in which applicants were asked about prior job experiences in more detail with particular emphasis on the administration of the SCID‐5; and 1‐h virtual interviews conducted by MDPS staff and expert consultants consisting of a standardized series of questions to assess facility with the SCID‐5 and research experience. CI applicants completed the interview with a roleplay exercise to demonstrate clinical interviewing skills and probing techniques. CS applicants completed a roleplay exercise to assess supervisory skills including providing feedback to team members.

Additional efforts were made to recruit applicants with more extensive training and expertise to fill the CS roles. Specifically, CS applicants were required to have a background in leading teams including the provision of clinical supervision to trainees (e.g., graduate students, interns, postdoctoral fellows).

The MDPS team hired 68 CIs and 11 CSs. In the summer of 2021, RTI recruited and hired 26 additional CIs to account for CI attrition. All CSs held a doctoral degree in psychology or social work. CIs included graduate students in APA‐accredited clinical and counseling psychology PhD programs, masters’‐level social workers, and masters’‐level researchers in mental health or a related field.

#### 4.2.2 Clinical interviewer and supervisor training

The clinical interview portion of the training program was developed, reviewed, and approved by developers of the SCID. Self‐paced pretraining content included recorded presentations on the Distressed Respondent Protocol, assessment of cognitive status using the Short Blessed Test, MDPS Clinical Interviewer Handbook, and User Guide for the SCID‐5‐NSMH. All CIs and CSs completed the pretraining content prior to MDPS training. The first MDPS training was a pilot training that occurred in the fall of 2020 with five CIs over a 1‐week period (five consecutive 8‐h training days). The pilot training was used to refine the subsequent main training. In the main training, the remaining CIs and CSs attended a 10‐day training (half‐day, 4‐h sessions), all delivered virtually. Additional “office hours” were offered to troubleshoot programming and equipment issues and to answer substantive questions from the training sessions. The training included a mix of live didactic presentations, watching and scoring prerecorded interviews with live question‐and‐answer sessions, paired mock exercises, and small group discussions using virtual breakout rooms. Experts moved between breakout rooms to engage in group discussions and provided personalized feedback to interviewers and supervisors in real time. Training also focused on the use of the computerized version of the SCID‐5‐NSMH.

In addition to the CI pretraining and training activities, CSs attended a 2‐h training on giving effective feedback, leading Quality Circle meetings, and the certification process. Bilingual interviewers attended additional review sessions in Spanish and were evaluated on their ability to complete the MDPS clinical interview in Spanish and English.

#### 4.2.3 Certification process

Upon successful completion of interviewer training, all CIs were required to pass a NetSCID certification process. The process consisted of two phases, both of which involved full video reviews of clinical interviews. Recorded certification interviews were evaluated and scored by their CS with a 33‐item rubric assessing CI performance in four domains: Interviewing Style, Obtaining Diagnostic Information, Skills Assessing Specific Disorders, and NetSCID Technical Skills. In Phase 1, CIs completed clinical interviews with volunteer respondents and roleplay activities with their CS. In Phase 2, CIs completed interviews with actual MDPS respondents. CIs were given up to three opportunities (separate certification interviews) to pass Phase 2.

All hiring and training was conducted virtually because of the COVID‐19 pandemic.

## 5 QUALITY CONTROL

Throughout data collection, CIs participated in a range of quality control (QC) activities. All MDPS interviews were recorded unless the respondent declined to be recorded. Respondents who declined were still able to complete the interview. QC tools included a scoresheet with ratings and interviewer notes to justify ratings and the video recording (split screen showing the faces of the interviewer and respondent). QC processes included (1) video recordings of 10% of all completed interviews evaluated using the standardized scoresheet, (2) partial video or clinical interview scoresheet note review of completed interviews when the CI requested help with a difficult case, (3) weekly CI Quality Circle supervision meetings, (4) weekly CS Quality Circle meetings, and (5) quarterly calibration exercises with scoresheet reviews to monitor inter‐rater reliability with retraining as needed to ensure consistency across all CIs. Spanish interviews underwent a peer‐review process in which bilingual CIs volunteered to review and report findings to their respective CS.

### 5.1 Full video review

CSs conducted full video reviews on 10% of completed clinical interviews. Cases were randomly selected from each CIs completed interviews for review. CSs utilized a standardized document (same rubric used in the certification process) and provided this form to the CI as part of the feedback process. CSs identified and corrected any coding errors (e.g., coding a symptom positively when the symptom did not meet the clinical threshold). After the completion of a full video review, the interview received one of three ratings: (1) no problems (no changes required by the CS), (2) minor problems (minor changes required by the CS at the item/symptom level resulting in no loss of data; all data at the diagnosis level were retained), and (3) major problems (changes required by the CS at the item or diagnosis level resulting in the loss of data; data coded as missing at the diagnosis level for at least one module). Interviews with major problems were retained, but affected modules were coded as missing data. When a full review indicated major problems, CSs would conduct partial reviews of other CI interviews to determine if the error occurred systematically across interviews or if it was an isolated error. The CS then met with the CI to provide feedback and retraining exercises (e.g., roleplay exercises, didactic refresher, develop action plan to prevent future errors). CSs frequently reviewed modules in the next interview to ensure that feedback was successfully implemented.

Across the interviews that received a full video review, 63% were rated with no problems, 33% with minor problems, and 4% with major problems.

### 5.2 Partial video or note review

When needed, CIs identified questions or modules for their CS to review. Upon completion of the CS review, the CI and CS discussed the item(s) in question and came to an agreement on a final rating. If uncertainty remained, the CS reviewed the issues with the CS supervisor and with one of the study's multiple PIs. Occasionally uncertainty persisted, and in these cases the issue was discussed with the developer of the SCID‐5 and editor of DSM‐5 TR (American Psychiatric Association, 2022), who was one of the study co‐investigators.

### 5.3 Quality Circle meetings and calibration exercises

Quality Circle meetings were regularly scheduled meetings where CIs met as a group with their assigned CS and received feedback on clinical topics that emerged from interviews conducted in the prior week. In addition, Quality Circle meetings were regularly scheduled for all CSs to meet as a group to discuss topics discussed in CI Quality Circle meetings. Quality Circle meetings were also used to review and discuss calibration exercises. Calibration exercises occurred on a quarterly basis in which all CSs and CIs watched the same clinical interview video recording (typically two to three diagnostic modules in length) and completed the NetSCID. NetSCID ratings were automatically sent to study statisticians. Across all raters, agreement across all CIs on calibration exercises at the diagnostic level was consistently greater than 90%.

## 6 RESPONSIVE SURVEY DESIGN

The MDPS study used a responsive survey design (RSD). Survey designs are typically informed by past survey experiences and experimentation. However, there is substantial variation in outcomes across surveys and, relatedly, in what design choices are best for any given survey. For the MDPS, many potential survey design choices had not been tested before or had not been used in the MDPS populations of interest. RSD is helpful because it acknowledges these uncertainties. Instead of fixing the study design for the entire data collection, RSD allows multiple protocols to be created and fielded in parallel (Groves & Heeringa, 2006). Results from each condition are monitored, and decisions are made on what design features to use for the later phases of data collection. The MDPS relied on RSD in two specific situations that will receive further attention in future publications: the design of the contact materials and the type of screening instrument.

One MDPS RSD experiment varied the return address sender of the household roster invitation letter (three versions: the sponsoring government agency, the survey organization, or the MDPS study branding) and the design of the household roster invitation letter (two versions: a standard letter design or a visual graphic design that illustrated the flow of respondent tasks). These six conditions were implemented in the early sample releases, and RRs were monitored. Based on the RRs, the experiment was stopped, and future releases only included the MDPS study branding in the return address and the visual graphic letter design as higher roster RRwere observed in those two conditions.

Another RSD feature of the MDPS design included the household screening instruments and stratification process. An effective screening instrument in this design would be one that accurately assigns respondents to their appropriate disorder sampling strata for efficient sampling into the clinical interview stage. One approach is to use items from a non‐adaptive instrument that administers the same items to each respondent. An alternative approach is to use an adaptive instrument that generates each subsequent question based on responses to the prior question. These approaches are described in the instrumentation section. In the MDPS, households were randomly assigned to the non‐adaptive or adaptive screening instrument for online administration. Here as well, results were monitored throughout data collection. In this case the decision was made to use early results to make minor adjustments to the stratum definitions (see prior *Household Sampling* description) and to continue using both screeners to collect more data to inform future similar study implementation efforts.

## 7 STATISTICAL WEIGHTING AND ANALYTIC PLAN

In this section we describe the process for generating MDPS analysis weights, methods for estimating the design effects (deffs), and the analytic plan to estimate national prevalence and treatment rate estimates.

### 7.1 Methods to account for nonresponse bias

RRs have been declining in household surveys (e.g., Williams & Brick, 2018). Although meta‐analyses of nonresponse bias studies have not shown a link between RR and nonresponse bias (Groves, 2006; Groves & Peytcheva, 2008), lower RR reduce the analytic utility of the data and increase the potential for nonresponse bias. To reduce nonresponse we utilized incentives at each stage of data collection, multiple modes of data collection (web, telephone, mail, in person, and virtual), and multiple contact attempts using varied materials and modes. The in‐person household data collection protocol was used for a random subset of nonresponding households in the early sample release replicates—approximately one‐third of the sample—to further help reduce the risk of nonresponse bias by focusing this more costly method on a subset of the sample and giving those randomly chosen households larger weights in analysis (i.e., nonresponse follow‐up [NRFU] methodology; see Chap. 17 in Valliant et al., 2018).

Nonresponse could occur in any of the three stages of data collection. The primary evaluation for roster nonresponse was external estimates from sources such as the American Community Survey (ACS). Selection‐weighted demographic distributions for randomly selected adults were compared to population distributions from the ACS 2020 5‐Year file (U.S. Census Bureau. 2021). Table 5 shows these estimates from MDPS and the ACS for several variables for the households, household members, and selected household members. The differences were small, 1.1% points on average, despite several being statistically significant. To minimize the impact of these differences between the MDPS sample and national population on national estimates, these demographic characteristics were used in the weighting adjustments described in the next section.

**TABLE 5:** Selection‐weighted demographic distributions for roster respondents and population estimates from the American Community Survey 5‐year estimates.

Unit of measurement	Variable	Category	MDPS roster respondents		American Community Survey	Difference	Significance
			%	Standard error	%	Percentage points	*p*‐Value^a^
Household	Region	Northeast	15.1	(1.0)	17.6	−2.5	0.014
		Midwest	23.4	(1.2)	22.1	1.3	0.259
		South	36.1	(1.5)	37.8	−1.7	0.258
		West	25.3	(1.5)	22.5	2.8	0.056
	Urbanicity	Urban	70.9	(4.2)	72.2	−1.3	0.756
		Urban cluster	11.4	(2.5)	9.3	2.0	0.410
		Rural	17.7	(2.5)	18.5	−0.8	0.768
	Household size	1	27.9	(0.6)	28.3	−0.4	0.490
		2	34.4	(0.5)	34.3	0.1	0.801
		3	15.1	(0.3)	15.3	−0.2	0.579
		4	12.9	(0.3)	12.5	0.4	0.187
		5+	9.7	(0.4)	9.6	0.1	0.859
	Tenure	Owner‐occupied	62.6	(1.3)	64.2	−1.5	0.249
		Renter‐occupied	35.7	(1.3)	34.1	1.6	0.248
		Other	1.7	(0.1)	1.7	0.0	0.851
		Child in household	29.1	(0.6)	29.9	−0.8	0.220
		Eligible adult	78.2	(0.8)	80.9	−2.7	0.000
Household member	Age	18–35	39.8	(0.7)	38.3	1.5	0.033
		36–49	27.8	(0.4)	28.6	−0.9	0.017
		50–65	32.5	(0.6)	33.1	−0.6	0.322
		Female	52.8	(0.2)	50.8	2.0	0.000
Selected person	Age	18–35	38.3	(0.7)	38.3	0.0	0.988
		36–49	28.8	(0.4)	28.6	0.2	0.587
		50–65	32.9	(0.7)	33.1	−0.2	0.768
		Female	53.2	(0.3)	50.8	2.4	0.000

^a^ *p*‐values obtained from weighted *t*‐tests calculated with estimates obtained from SUDAAN's CROSSTAB procedure (Research Triangle Institute, 2012, chap. 14) to account for the complex MDPS design.

Questions were included in the MDPS household roster with the objective to evaluate and adjust for nonresponse at the screener stage. This information is available for both respondents and nonrespondents to the screener. All differences were below 1% point except for one—emotional problems (Table 6). Reports for anyone with emotional problems in the household were 6% points lower among nonrespondents (31.8%) compared to screener respondents (37.8%; *p* < 0.000; see Table 6). This difference was not explained by differences in demographic characteristics (results not shown). Based on these findings, this variable was included in weighting adjustments for screener nonresponse.

**TABLE 6:** Selection‐weighted roster estimates for screener respondents and nonrespondents, for six conditions.

	Screener respondents		Screener nonrespondents		Difference	Significance
	Percent	Standard error	Percent	Standard error	Percentage points	*p*‐Value^a^
Diabetes	16.9	(0.6)	18.9	(0.7)	−1.9	0.003
Heart problems	14.7	(0.5)	15.2	(0.6)	−0.5	0.434
Cancer	9.0	(0.3)	9.1	(0.4)	−0.1	0.776
Schizophrenias	2.3	(0.2)	2.4	(0.2)	−0.1	0.696
Emotional problems	37.8	(0.8)	31.8	(0.9)	6.0	0.000
Alcohol/drug problems	10.1	(0.4)	9.3	(0.4)	0.8	0.063

^a^ *p*‐values obtained from weighted chi‐square tests calculated via SUDAAN's CROSSTAB procedure (Research Triangle Institute, 2012, chap. 14) to account for the complex MDPS design.

The screener, in turn, collected information on mental health and substance use. This information is then available for respondents and nonrespondents to the clinical interview, providing a rich set of variables for evaluation and correction for nonresponse occurring at the third stage in household data collection. Ten CIDI screener outcome measures and nine CAT‐MH screener outcome measures (the CAT‐MH combines alcohol abuse and drug abuse into substance abuse) were used to evaluate clinical interview nonresponse. Respondents and nonrespondents were significantly different on 10 of the 19 measures (Table 7). Nonresponse bias was also estimated (the difference between weighted estimates calculates from the clinical interview respondents and the full clinical interview sample). The average nonresponse bias was only 1.7% points, and that average was driven by two extreme values. The nonresponse bias for the CIDI and CAT‐MH Moderate Health Disability was 6.3% and 6.7%, respectively. These variables were used in the clinical interview nonresponse weighting adjustments.

**TABLE 7:** Selection‐weighted screener estimates for clinical interview respondents and nonrespondents, for 10 CIDI measures and nine parallel CAT‐MH measures.

Variable	Category	Respondents (*n* = 4732)		Nonrespondents (*n* = 8043)			
		CIDI (*n* = 3485)		CIDI (*n* = 5809)			
		CAT‐MH (*n* = 1247)		CAT‐MH (*n* = 2234)		Difference	Significance
		%	Standard error	%	Standard error	Percentage points	*p*‐Value^a^
CIDI depression	Yes	12.5	(0.7)	10.8	(0.5)	1.7	0.026
CIDI anxiety	Yes	10.8	(0.6)	9.4	(0.4)	1.3	0.072
CIDI mania/hypomania	Yes	24.7	(1.3)	21.9	(0.8)	2.9	0.057
CIDI PTSD	Yes	34.6	(1.3)	29.9	(1.1)	4.6	0.004
CIDI alcohol abuse	Yes	2.6	(0.4)	2.5	(0.2)	0.1	0.793
CIDI drug abuse	Yes	4.9	(0.5)	5.4	(0.4)	−0.5	0.407
CIDI psychosis—Severe	High	12.8	(0.8)	11.9	(0.5)	1.0	0.058
CIDI psychosis—Moderate	Moderate	3.0	(0.4)	2.1	(0.2)	1.0	
CIDI Health/disability—Severe	High	1.7	(0.2)	2.5	(0.3)	−0.8	0.000
CIDI health/disability—Moderate	Moderate	34.5	(1.4)	25.3	(1.0)	9.2	
CAT‐MH depression	Yes	12.0	(1.1)	8.2	(0.6)	3.8	0.001
CAT‐MH anxiety	Yes	19.7	(1.4)	17.0	(1.0)	2.7	0.102
CAT‐MH mania/hypomania	Yes	9.7	(1.2)	9.0	(0.8)	0.7	0.602
CAT‐MH PTSD	Yes	5.9	(0.8)	3.6	(0.4)	2.3	0.013
CAT‐MH substance abuse	Yes	12.4	(1.1)	10.8	(0.8)	1.6	0.202
CAT‐MH psychosis—Severe	High	1.5	(0.5)	1.1	(0.2)	0.4	0.424
CAT‐MH psychosis—Moderate	Moderate	3.7	(0.6)	3.0	(0.3)	0.7	
CAT‐MH health/disability—Severe	High	2.3	(0.7)	1.3	(0.3)	1.0	0.000
CAT‐MH health/disability—Moderate	Moderate	36.6	(2.1)	27.2	(2.1)	9.4	
Combined depression	Yes	12.4	(0.6)	10.1	(0.4)	2.3	0.001
Combined anxiety	Yes	13.0	(0.6)	11.5	(0.5)	1.5	0.046
Combined mania/hypomania1	Yes	20.9	(0.9)	18.3	(0.7)	2.6	0.018
Combined PTSD	Yes	27.3	(0.9)	22.5	(0.8)	4.7	0.000
Combined substance abuse	Yes	8.3	(0.5)	8.3	(0.4)	0.0	0.990
Combined psychosis	High	9.9	(0.6)	8.8	(0.4)	1.1	0.022
Combined psychosis	Moderate	3.2	(0.3)	2.3	(0.2)	0.9	
Combined health/disability	High	1.9	(0.3)	2.2	(0.2)	−0.3	0.000
Combined health/disability	Moderate	35.1	(1.1)	25.8	(1.0)	9.2	

^a^ *p*‐values obtained from weighted chi‐square tests calculated via SUDAAN's CROSSTAB procedure (Research Triangle Institute, 2012, chap. 14) to account for the complex MDPS design.

With the revised weights, subsequent analyses confirmed that the adjustments were useful in limiting detectable levels of nonresponse bias at each stage that could impact the generalizability of the household estimates.

### 7.2 Weighting

The goal of the MDPS was to produce national estimates for U.S. adults ages 18–65. To produce such estimates, analysis weights were developed to account for the complex sampling design with adjustments to limit nonresponse bias.

Population estimates were produced from survey‐weighted data with software that accounted for the complex MDPS sampling design (see, e.g., Valliant & Dever, 2018). Survey weights were calculated independently for each of the four population samples that reflected the relative sizes of the age‐eligible adult population and combined to form the MDPS analysis weights as discussed below.

*Creation of Household Weights*. For the household weights, respondent records were weighted inversely to the selection probability—address, NRFU subsample, persons within household, and screener respondents for the clinical interview; adjusted for nonresponse to the screener using frame (region, children in household, household size, homeownership status) and roster (roster mode, household health items) data and to the clinical interview using frame, roster, and screener (clinical interview strata, disorder indicators, screener mode, inclusion in NRFU); and then poststratified to 2019 1‐year ACS estimates for the frame items and demographics (age group, race/ethnicity, sex at birth, highest education, marital status). The final set of model covariates was adjusted to limit bias detected in the nonresponse bias evaluation as discussed previously.

*Creation of Non‐household Weights*. The *prison weights* included components for selection of facility and persons within facility and for poststratification to BJS national estimates of inmates in 2020 by sex at birth, race/ethnicity, and age group (BJS, 2021). The *hospital convenience weights* included a within‐facility person‐level selection probability and a poststratification adjustment by state and overall to the 2018 national estimates provided by the National Association of State Mental Health Program Directors (Lutterman, 2022). Similarly, the *shelter convenience weights* reflect the inverse of the within‐shelter selection probability and a by‐state poststratification to the 2021 U.S. Housing and Urban Development (2023) Point‐in‐Time estimates. All nonresponse and poststratification components were derived with the WTADJUST procedure in SUDAAN® (Research Triangle Institute, 2012). Missing responses for the weighting variables were replaced with data obtained from a corresponding instrument (e.g., household roster for missing screener data) or through weighted hot‐deck imputation via SUDAAN's IMPUTE procedure.

### 7.3 Creation of MDPS total population analysis weights

The independently generated sample‐specific analysis weights were adjusted to the respective populations through, for example, poststratification to available population characteristics. Consequently, no further adjustment was required for analysis of the full data file as the weights reflect the relative size of each population among the four populations included in MDPS. For example, a prevalence estimate for disorder *d* among U.S. adults 18–65 years of age living in household *and* the specific non‐household settings of interest to MDPS is defined as:

$${\hat{p}}_{d}=\frac{\sum_{h=1}^{4}\sum_{s_{h}}w_{hi}\delta_{hid}}{\sum_{h=1}^{4}\sum_{s_{h}}w_{hi}}$$

where sample *s*_*h*_ from population *h*, person *i*, person‐level analysis weights *w*_*hi*_, and binary (0–1) indicator for the presence of disorder *d I*_*hdi*_ (see sec. 7.4 of Valliant & Dever, 2018). Note that because the population‐specific analysis weights were scaled to the size of mutually exclusive populations discussed below, *W*_*h*_ = 1 for all *h*. Consequently, information from adult participants living in residential households makes up 99.2% of the MDPS target population and associated prevalence estimates. The remaining 0.8% of the combined MDPS target population includes the federal/state prisons population (0.6%), the state psychiatric hospital population (0.02%), and the sheltered homeless population (0.2%).

### 7.4 Design effects of the survey weights

The survey weights were designed to produce estimates generalizable to the U.S. adult population ages 18–65. However, MPDS utilized complex features in design and weighting, such as cluster sampling design for the household sample, NRFU subsampling, nonresponse and poststratification weighting adjustments, and oversampling of certain groups at the clinical interview stage and among non‐household populations. These features were designed and incorporated for different purposes to enhance the overall quality of the results within some practical constraints (e.g., cost, time, and feasibility to collect data). However, many of the components can increase the magnitude of the deff compared to a simple random sample with the same sample size. The deff—the ratio of the weighted variance of an estimate accounting for all sampling design features to the unweighted variance that ignores the design (i.e., a simple random sample)—is used to measure the impact of the sampling design and weighting adjustments on the precision of estimates compared to a simple random sample of the same size (Valliant et al., 2018). The median deff for the eight MDPS mental disorders was 3.7 for the combined sample and 3.2 for the residential household sample alone. The median deff across the five MDPS SUDs was 2.9 for the combined sample and 2.6 for the household sample. Constraints to limit the variability of the weights were evaluated but discarded when the population prevalence estimates were negatively affected.

Population estimates and their design‐based (i.e., Taylor Series) standard errors can be calculated with the MDPS data using the weights, the sampling variables, and software that accounts for the complex sampling design such as SUDAAN. Weighted prevalence estimates overall and by select characteristics along with 95% confidence intervals for key MDPS mental and SUDs are published elsewhere (Ringeisen et al., 2023).

## 8 STUDY RECRUITMENT RESULTS

The unweighted sample counts and weighted conditional RR for each of the MDPS study populations and stage have been published in the *MDPS: Findings Report* (Ringeisen et al., 2023). In the household sample, The American Association for Public Opinion Research (AAPOR) (2023) RR3 formula and base weights were used for the weighted roster RR calculation. The AAPOR (2023) RR1 formula and person‐level base weights adjusted for NRFU were used for the weighted screener and clinical interview RR calculations. In the prison sample, the AAPOR (2023) RR1 formula and person‐level base weights adjusted for NRFU were used for the weighted institution (prisons) RR whereas the AAPOR (2023) RR1 formula and person‐level base weights were used for the weighted clinical interview RR. RRs were not calculated for the homeless shelter or state psychiatric hospitals, nor was an overall RR calculated for the full sample given the differences in the sampling and recruitment approaches.

### 8.1 Household sample count and response rates

Figure 1 shows the sample counts and weighted RRs for the residential household sample at each stage of recruitment and data collection. For example, among the 234,270 sampled households, there were 25,752 completed rosters, for a weighted RR of 17.4% (AAPOR, 2023 RR3 formula). Among the 41,868 adults aged 18–65 randomly chosen from 25,752 rosters, 29,084 completed the screening interview, resulting in a weighted RR of 67.4% (AAPOR, 2023 RR1 formula) among roster respondents. The overall weighted screening interview RR, accounting for the roster stage, was 11.7%. Among the 12,906 adults with a completed screening interview who were randomly selected for the next phase of the study, 4764 adults completed the clinical interview for a 31.2% weighted RR (AAPOR, 2023 RR1 formula). The overall weighted household clinical interview RR, accounting for the prior stages, was 3.7%. The lower than expected overall RR is consistent with the impact of the COVID‐19 pandemic (Krieger et al., 2023; U.S. Bureau of Labor Statistics, 2022; U.S. Census, 2022) and other societal changes on RR in other surveys (Brick & Williams, 2013; Tolonen et al., 2006; Williams & Brick, 2018).

### 8.2 Household sample characteristics

Table 8 displays the characteristics of the participants from the household sample at each stage of data collection and the weighted percentages. For example, among the 29,084 adults who completed the screening interview, 14.9% were aged 18–25; 50.9% were female at birth; 18.2% reported being of Hispanic or Latino origin; 48.9% were married; 26.1% had a high school degree or equivalent; 63.6% owned their home; and 37.9% lived in the southern region of the United States. As anticipated, most participants (53.2%) were classified into “no reported symptoms associated with mental or substance use disorders” (sampling stratum 3); 10.4% were classified into sampling stratum 1 who indicated report of possible psychotic symptoms or disability payments associated with schizophrenia.

**TABLE 8:** Sociodemographic characteristics of MDPS participants from randomly chosen residential households.

Sociodemographic characteristics	Selected for					
	Screening interviews weighted		Clinical interview weighted		Completed clinical interview weighted	
	Count^a^	Percent^b^	Count^a^	Percent^c^	Count^a^	Percent^d^
Overall	29,084	100.0	12,916	100.0	4764	100.0
Age						
18–25	3714	14.9	1972	18.0	610	16.8
26–44	13,326	44.4	5993	44.4	2216	42.0
45–65	12,044	40.8	4951	37.6	1938	41.2
Sex at birth						
Male	12,241	49.1	5160	47.3	1804	48.9
Female	16,843	50.9	7756	52.7	2960	51.1
Current gender identity^e^						
Male					1769	48.3
Female					2888	50.5
Transgender/gender diverse					75	0.9
Race/ethnicity						
Hispanic/Latino	5410	18.2	2409	17.9	712	18.3
White, not Hispanic	17,258	59.0	7598	58.4	3086	59.7
Black/African American, not Hispanic	3028	12.4	1433	12.9	457	12.5
Asian, not Hispanic	1841	4.9	707	4.3	262	4.4
AIAN or NHPI, not Hispanic^f^	352	1.3	181	1.5	47	0.6
Multiracial, not Hispanic	888	3.1	464	4.0	198	4.4
Marital status						
Married	13,956	48.9	5245	41.3	1993	49.1
Widowed/divorced/separated	4900	14.3	2429	16.1	870	14.4
Never married	10,031	36.1	5163	41.9	1886	36.1
Highest education						
Less than high school	1849	10.2	943	11.5	187	10.4
High school (equivalent)	5257	26.1	2614	28.6	644	26.5
Some college	5749	21.3	2852	23.1	934	21.3
Bachelor's degree	10,098	30.3	4232	27.3	1825	30.4
Advanced degree	5920	11.1	2187	8.7	1157	11.2
In college/university						
Yes	1672	4.3	754	4.4	342	3.6
No	27,219	95.0	12,073	94.9	4406	96.0
Employment status						
Yes	19,283	61.7	7983	56.4	3063	59.9
No/retired	9536	37.3	4818	42.7	1678	39.6
Military veteran						
Yes	1675	6.0	736	6.0	290	6.7
No	27,218	93.3	12,096	93.3	4455	93.0
Household tenure						
Own home	17,439	63.6	7032	58.8	2770	63.6
Rent/other	11,645	36.4	5884	41.2	1994	36.4
Income^e^	‐	‐	‐	‐		
Less than $20,000					719	15.7
$20,000–$49,999					1005	23.9
$50,000–$74,999					729	13.7
$75,000–$99,999					591	10.2
$100,000–$149,999					662	12.3
$150,000 or more					756	14.8
Region						
Midwest	6233	20.5	2739	20.1	1108	20.7
Northeast	4354	17.3	1887	16.9	748	17.3
South	9675	37.9	4477	39.2	1607	38.0
West	7347	24.2	3272	23.9	1301	24.1
Urbanicity^c^						
Urban	24,575	80.6	10,975	82.1	4092	82.0
Rural	4509	19.4	1941	17.9	672	18.0
Disorder strata						
1‐Possible psychosis	2856	10.4	2856	23.7	1097	10.2
2‐Possible other conditions	11,193	36.4	7412	55.9	2858	39.3
3‐No conditions detected	15,035	53.2	2648	20.4	809	50.5

^a^ Unweighted participant counts. Counts by characteristics may not sum to the total because of nonresponse to the questionnaire item.

^b^ Percent calculated with screening interview analysis weights.

^c^ Percent calculated with screening interview analysis weights adjusted for clinical interview subsampling.

^d^ Percent calculated with clinical interview analysis weights.

^e^ Gender identity and income asked only during the clinical interview.

^f^ Includes American Indian/Alaskan Native (AIAN) and Native Hawaiian/Other Pacific Islander (NHPI), not Hispanic persons.

Among the screening interview participants displayed in the second set of columns in Table 8, a total of 12,916 adults were randomly selected for the clinical interview. The clinical interview sample weighted distributions were roughly comparable to the weighted distributions for the screening interview participants. As discussed, all participants in sampling stratum 1 (“psychosis” risk) were asked to complete a clinical interview; moderate and low sampling rates were used for strata 2 and 3, respectively.

As shown in the last set of columns in Table 8, a total of 4764 household participants completed clinical interviews. Among these participants, 41.2% were 45–65 years old; 48.9% were male at birth; 12.5% reported their race as Black/African American; 3.6% reported taking courses at college or university; and 6.7% described themselves as military veterans. Almost 1100 participants classified into sampling stratum 1 were interviewed (1097; 10.2%), along with 2858 (39.3%) adults from sampling stratum 2 and 809 (50.5%) adults from sampling stratum 3.

### 8.3 Non‐household sample counts and response rates

Figure 2 shows the flowchart for the state/federal prison sample. Among the 50 prisons randomly chosen for MDPS, 22 participated for a 43.5% weighted RR. A total of 606 prison residents were randomly selected from roster information provided by the participating facilities, and 321 (49.6% weighted RR) completed the clinical interview.

Figure 3 contains the sample counts for the two remaining non‐household samples: (1) the state psychiatric hospital sample in panel A, and (2) the homeless shelter sample in panel B. Selected agencies may have had administrative responsibility for multiple facilities. Forty‐six percent of homeless shelters and 36% of state psychiatric hospitals contacted for the study agreed to participate. A total of 1315 homeless shelter residents were randomly selected from roster information provided by the participating facilities, and 423 (32.2% weighted RR) completed the clinical interview. A total of 646 hospital residents were randomly selected from roster information provided by the participating facilities, and 171 (26.5% weighted RR) completed the clinical interview.

The primary reasons for non‐response among the sample selected within the non‐household facilities were: refusal or breakoff (did not complete the clinical interview), not available at the time of the interview (i.e. working, offsite), no longer at the facility at the time of the interview, mental capacity, or refusal by the facility.

### 8.4 Non‐household sample characteristics

Table 9 displays the characteristics (counts and weighted percentages) of the participants from the non‐household probability‐based (state/federal prisons) and convenience (psychiatric hospital and homeless shelter) samples. From the randomly chosen state/federal prisons, 321 inmates completed the clinical interview including 11.9% were 18–25 years old; 6.9% were female at birth, 59.6% reported that they were never married, and 29.9% reported having less than a high school education.

**TABLE 9:** Sociodemographic characteristics of MDPS clinical interview participants from the three non‐household samples.

Sociodemographic characteristics	State/fed prisons weighted		Psychiatric hospitals weighted		Homeless shelters weighted	
	Count^a^	Percent^b^	Count^a^	Percent^b^	Count^a^	Percent^b^
Overall	321	100.0	171	100.0	423	100.0
Age						
18–25	24	11.9	27	15.1	30	11.2
26–44	209	58.4	89	42.0	180	43.3
45–65	88	29.8	55	42.9	213	45.5
Sex at birth						
Male	211	93.1	128	73.4	228	54.6
Female	110	6.9	43	26.7	195	45.4
Race/ethnicity						
Hispanic/Latino	56	33.6	24	33.2	90	33.1
White, not Hispanic	143	25.3	81	26.6	141	20.2
Black/African American, not Hispanic	81	27.7	39	28.0	129	28.4
Other/multiracial, not Hispanic^c^	39	13.0	17	6.8	42	9.0
Marital status						
Married	28	10.4	10	2.4	22	5.8
Widowed/divorced/separated	93	27.2	50	24.5	138	29.5
Never married	190	59.6	105	68.8	243	58.3
Highest education						
Less than high school	77	29.9	47	33.8	81	15.0
High school (equivalent)	152	50.9	53	35.6	158	41.4
Some college	53	11.5	38	16.1	88	19.2
Bachelor's or advanced degree	29	4.9	28	10.2	76	17.7
Employment status						
Yes	169	45.0	54	10.7	86	21.6
No/retired	140	51.3	111	85.0	317	71.9
Region						
Midwest	84	15.6	0	0.0	0	0.0
Northeast	26	8.5	44	80.5	206	83.0
South	147	52.0	57	13.9	79	5.6
West	64	23.8	70	5.6	138	11.4

^a^ Unweighted participant counts. Counts by characteristics may not sum to the total because of nonresponse to the questionnaire item.

^b^ Percent calculated with clinical interview analysis weights.

^c^ Includes non‐Hispanic race/ethnicity categories of Asian, American Indian/Alaskan Native, Native Hawaiian/Other Pacific Islander, and multiracial.

From the recruited psychiatric hospitals, 171 residents completed the clinical interview among 646 randomly selected residents. Of those who completed a clinical interview, 15.1% were 18–25 years old, 26.7% were female at birth, 68.8% reported having never married, 33.8% reported having less than a high school education, and 10.7% reported being currently employed.

A total of 1315 homeless shelter residents were recruited for the clinical interview, resulting in 423 completed interviews. Of those who completed a clinical interview, 11.2% were 18–25 years old, 45.4% were female at birth, 58.3% reported having never married, 41.4% reported having a high school diploma or equivalent, and 71.9% reported being either not employed or retired.

## 9 DISCUSSION

The MDPS is designed to assess prevalence rates of specific mental and SUDs among adults ages 18–65 in the United States and the proportion of individuals with these disorders who received any treatment in the past year. The MDPS screening and clinical interview data will be made available for restricted use upon approval through the Inter‐university Consortium for Political and Social Research (https://www.icpsr.umich.edu/), including user documentation and instruments, and will provide rich opportunities for analyses. The anticipated release is in early 2024.

In contrast to past epidemiological studies of mental and SUDs, the MDPS emphasized the assessment of SSDs. This guided all aspects of the study design, sometimes requiring novel approaches. Although SSDs are typically chronic disorders, they may remit or relapse, especially in response to treatment or non‐adherence to treatment. To provide an estimate of all individuals with SSDs, even those in remission or partial remission, the MDPS assessed both lifetime and past‐year prevalence of SSDs. Because of interview time constraints, for all other disorders the MDPS assessed only past‐year prevalence.

SSDs are uncommon, with most previous studies estimating lifetime prevalence to be less than 1% (Desai et al., 2013; Kessler, Berglund, et al., 2005; Moreno‐Küstner et al., 2018; Saha et al., 2005; Wu et al., 2006). To increase the proportion of clinical interviews with individuals with severe mental health disorders, the MDPS utilized a multistage design that first screened for individuals at higher risk of SSDs and other mental or SUDs. These individuals were then oversampled for clinical interviews. Along with enhancing the precision of prevalence estimates, this multistage design also increased the number (unweighted) of individuals in the MDPS sample with mental and substance disorders 2‐ to 3‐fold, increasing the statistical power for subgroup analysis.

Fully structured interviews administered by lay interviewers are not well suited for the assessment of SSDs, because such assessment often requires considerable clinical judgment. Researchers have utilized versions of the fully structured Composite International Diagnostic Interview (Kessler & Ustün, 2004), conducted by lay interviewers, to estimate the prevalence and predictors of psychosis (Jacobi et al., 2014; Kessler, Berglund, et al., 2005; Lieb et al., 2000; Schmidt‐Kraepelin et al., 2015; Wittchen et al., 1998, 2011). These assess 2 of the 5 DSM 5 “A Criteria” for SSDs, delusions and hallucinations, but not negative symptoms such as disordered speech or motor behavior, which require clinical observation and judgment. For example, the NCS‐R utilized six fully structured questions with yes‐no response options that asked about the DSM‐IV delusions and hallucinations, found in the NCS (Kendler et al., 1996) to be the strongest predictors of clinician‐diagnosed non‐affective psychosis (NAP). Respondents who endorsed any of the NAP symptom questions were asked to describe instances of the endorsed symptoms. Lay interviewers probed for complete responses and recorded responses verbatim. Preliminary clinical review of these responses yielded a lifetime NAP prevalence estimate of 1.5%. In a subsequent clinical reappraisal study with a mental health professional administering the SCID, the estimated lifetime NAP prevalence was considerably lower, 0.3%. This illustrates the difficulty of using lay interviewers and fully structured interviews to estimate the prevalence of SSDs or NAP. The Early Developmental Stages of Psychopathology Study successfully employed CIs to assess mental health disorders utilizing the M‐CIDI, although in a population younger than that of the MDPS (Dominguez et al., 2010, 2011; Lataster et al., 2012; Smeets et al., 2012; Wigman et al., 2012). Both studies have shown that it is feasible to conduct population‐based studies to assess symptoms associated with psychotic disorders such as SSDs with clinicians interviewing community dwellers.

The SCID‐5, originally developed for use in clinical settings, is the gold standard for measuring mental and SUDs. In particular, the SCID‐5 allows clinician interviewers to probe on each symptom, using their clinical judgment, while still strictly adhering to DSM‐5 criteria. In clinical studies, the SCID‐5 is usually used to measure one, or at most a few, disorders. Even in these situations, the SCID‐5 administration may take well over an hour to administer. The MDPS assessed 11 disorders. Prior to initiating data collection, the MDPS team knew that the SCID‐5 would require streamlining to be feasible for use in household and non‐household settings.

The study team worked with the SCID‐5 developer (MBF) to shorten the SCID‐5 administration time while maintaining strict adherence to DSM‐5 criteria, overall rigor, and the essential format of the SCID‐5. The average administration time for the SCID‐5‐NSMH portion of the MDPS clinical interview was less than 1 h in the household and non‐household settings. In past studies it has not been feasible to send trained mental health clinicians to households to conduct clinical interviews. But with the advent of virtual interviewing and the streamlined SCID‐5‐NSMH, the MDPS team successfully launched a large‐scale data collection effort. This approach has marked advantages. The SCID‐5 is the gold standard for diagnostic assessment. It is particularly useful when clinical knowledge and judgment is important, such as when assessing SSDs, determining if a disorder is substance induced, or determining if a disorder is the result of a general medical condition.

A key objective of the MDPS study was to investigate methods for use in future studies, including testing whether MDPS data collection methods could be implemented with high quality in prisons, homeless shelters, and state psychiatric hospitals. Because of this objective, individuals from these facilities were significantly oversampled. Results showed that interview quality and rigorous data collection standards could be upheld in both household and non‐household settings. These settings are of particular importance because past research suggests that individuals residing in these facilities have significantly higher rates of mental and SUDs. Inclusion of these populations make the MDPS sample more representative of the U.S population.

## 10 LIMITATIONS

The MDPS needs to be understood in the context of its limitations. The first is the low overall RR. The overall RR was impacted by the lower than planned use of field interviewers to conduct household rostering because of COVID‐19 restrictions on in‐person data collection. Also, the overall RR was reduced by including a screening interview, which introduced an extra stage of data collection. The study team used nonresponse weights at each design stage to account for nonresponse bias. To decrease response burden, the MDPS focused narrowly on disorder prevalence and treatment use. As a result, the clinical interview did not include some other relevant outcomes of interest, such as age of disorder onset and lifetime prevalence (except for SSDs). Further, the MDPS clinical interview did not differentiate between schizophrenia and schizoaffective disorder, or schizoaffective disorder and schizophreniform disorder. To do so would have required an assessment of lifetime MDE, which was not done because of time constraints. The MDPS clinical interview did classify SSD with duration greater than 6 months (which includes schizophrenia and schizoaffective disorder), and SSD with duration 6 months or less (which includes schizophreniform disorder and schizoaffective disorder). A final limitation is that some MDPS study results may have limited generalizability outside of the historical context of the COVID‐19 pandemic. The pandemic likely impacted the prevalence rates of some disorders, particularly those where psychosocial stressors have an important causative role. The WHO estimates that the prevalence of anxiety and depression has increased 25% because of COVID‐19 (WHO, 2022).

## 11 CONCLUSION

The MDPS provides the most up‐to‐date prevalence estimates of specific mental disorders in the non‐elderly U.S. adult population and the proportion of individuals with disorders who receive treatment. Among psychiatric epidemiological studies conducted in the United States, its emphasis on SSDs is unique. The MDPS will be publicly available and will provide rich opportunities for future analyses.

## AUTHOR CONTRIBUTIONS

**Heidi Guyer**: Methodology; investigation; project administration; supervision; visualization; writing – original draft; writing – review & editing. **Heather Ringeisen**: Conceptualization; project administration; methodology; investigation; visualization; writing – original draft; writing – review & editing. **Jill Dever**: Data curation; formal analysis; methodology; visualization; writing – original draft; writing – review & editing. **Dan Liao**: Formal analysis; methodology; visualization; writing – original draft; writing – review & editing. **Andy Peytchev**: Formal analysis; methodology; visualization; writing – original draft; writing – review & editing. **Christine Carr**: Methodology; supervision; validation; investigation; writing – original draft. **Paul Geiger**: Methodology; supervision; validation; investigation; writing – original draft. **Leyla Stambaugh**: Conceptualization; methodology; supervision; validation; investigation. **Tim Smith**: Methodology; supervision; validation; investigation. **Lisa Dixon**: Conceptualization; methodology; investigation. **Mark Olfson**: Conceptualization; methodology; writing – review & editing. **Michael First**: Methodology. **Scott Stroup**: Methodology. **Lydia Chwastiak**: Methodology; investigation. **Maria Monroe‐Devita**: Methodology; investigation. **Jeff Swanson**: Methodology; investigation. **Marvin Swartz**: Methodology; investigation. **Ronald C. Kessler**: Methodology; formal analysis; writing – review & editing. **Robert Gibbons**: Methodology; formal analysis. **Natalie Bareis**: Methodology; investigation; supervision. **Elizabeth Sinclair Hancq**: Conceptualization; methodology. **Thomas Clarke**: Conceptualization; funding acquisition; project administration; methodology; writing – original draft; writing – review & editing. **Mark Edlund**: Conceptualization; project administration; methodology; investigation; visualization; writing – original draft; writing – review & editing. **MDPS Consortium**: Conceptualization; methodology; investigation; supervision; validation; visualization; formal analysis; writing – original draft; writing – review & editing.

## CONFLICT OF INTEREST STATEMENT

The authors declare no conflicts of interest, with the exception of Dr. Gibbons. Dr. Gibbons has been an expert witness for the US Department of Justice, Merck, Glaxo‐Smith‐Kline, Pfizer and Wyeth and is a founder of Adaptive Testing Technologies, which distributes the CAT‐MH™ battery of adaptive tests. The terms of this arrangement have been reviewed and approved by the University of Chicago in accordance with its conflict of interest policies.

## DISCLAIMER

The views and opinions contained in this presentation do not necessarily reflect those of SAMHSA or the U.S. Department of Health and Human Services and should not be construed as such.

## MDPS AUTHOR AFFILIATIONS AND CONSORTIUM MEMBERS*

^1*^ **RTI International**: Heather Ringeisen, Mark Edlund, Heidi Guyer, Jill Dever, Dan Liao, Andy Peytchev, Christine Carr, Paul Geiger, Leyla Stambaugh, Tim Smith

^2*^ **Columbia University/New York State Psychiatric Institute**: Lisa Dixon, Mark Olfson, Scott Stroup, Thomas Smith, Michael First, Natalie Bareis

^3*^ **University of Washington**: Lydia Chwastiak, Maria Monroe‐DeVita, Mackenzie Tennison, Katherine Winans, Scott Graupensperger

^4*^ **Duke Health**: Marvin Swartz, Jeffrey Swanson, Allison Robertson

^5*^ **Harvard Medical School**: Ronald C. Kessler

^6*^ **University of Chicago**: Robert Gibbons

^7*^ **Treatment Advocacy Center**: Elizabeth Sinclair Hancq

^8^ **Substance Abuse and Mental Health Services Administration (SAMHSA)**: Thomas Clarke

## Data Availability

The MDPS screening and clinical interview data will be made available for restricted use upon approval through the Inter‐university Consortium for Political and Social Research (https://www.icpsr.umich.edu/), including user documentation and instruments, and will provide rich opportunities for analyses. The anticipated release is late 2023.
